# Perceptual processing links autism and synesthesia: A twin study

**DOI:** 10.1192/j.eurpsy.2021.367

**Published:** 2021-08-13

**Authors:** J. Neufeld, T. Van Leeuwen, L. Wilsson, H. Norrman, M. Dingemanse, S. Bölte

**Affiliations:** 1 Center Of Neurodevelopmental Disorders At Karolinska Institutet (kind), Karolinska Institutet, Stockholm, Sweden; 2 Donders Institute For Brain, Cognition And Behaviour, Radboud University, Nijmegen, Netherlands; 3 Centre For Language Studies, Radboud University, Nijmegen, Netherlands; 4 Child And Adolescent Psychiatry, Stockholm Health Care Services, Region Stockholm, Stockholm, Sweden; 5 Curtin Autism Research Group, Essential Partner Autism Crc, School Of Occupational Therapy, Social Work And Speech Pathology, Curtin University, Perth, Australia

**Keywords:** autism spectrum condition, synesthesia, twin design, sensory processing

## Abstract

**Introduction:**

Synesthesia is a non-pathological condition where sensory stimuli (e.g. letters or sounds) lead to additional sensations (e.g. color). It occurs more commonly in individuals diagnosed with Autism Spectrum Condition (ASC) and is associated with increased autistic traits and autism-related perceptual processing characteristics, including a more detail-focused attentional style and altered sensory sensitivity. In addition, autistic traits correlate with the degree of synesthesia (consistency of color choices on an objective synesthesia test) in non-synesthetes.

**Objectives:**

We aimed to investigate whether the degree of synesthesia for graphemes is associated with autistic traits and perceptual processing alterations within twin pairs, where all factors shared by twins (e.g. age, family background, and 50-100% genetics) are implicitly controlled for.

**Methods:**

We investigated a predominantly non-synesthetic twin sample, enriched for ASC and other neurodevelopmental disorders (n=65, 14-34 years, 60% female), modelling the linear relationships between the degree of synesthesia and autistic traits, sensory sensitivity, and visual perception, both within-twin pairs (22 pairs) and across the entire cohort.

**Results:**

A higher degree of synesthesia was associated with increased autistic traits only within the attention to details domain, with sensory hyper-, but not hypo-sensitivity and with being better in identifying fragmented images. These associations were stronger within-twin pairs compared to across the sample.

**Conclusions:**

Consistent with previous findings, the results support an association between the degree of synesthesia and autistic traits and autism-related perceptual features, however restricted to specific domains. Further, the results indicate that a twin design can be more sensitive for detecting these associations.

**Disclosure:**

No significant relationships.

